# Improving Outcomes for Community Substance Misuse Service Users Presenting in Acute Mental Health Crisis

**DOI:** 10.1192/bjo.2025.10428

**Published:** 2025-06-20

**Authors:** Kevin O’Neill, Munzir Quraishy

**Affiliations:** North London Mental Health Partnership, London, United Kingdom

## Abstract

**Aims:** Research shows that mental health problems are experienced by the majority of drug (70%) and alcohol (86%) users in community substance misuse treatment.

Islington Better Lives is a busy inner London community substance misuse clinic and service users frequently present in mental health crisis. The substance misuse multidisciplinary team is primarily nonmedical, as a result substance misuse keyworkers reported feeling uncertain of how to manage and at times overwhelmed by service users in acute mental health crisis.

**Methods:** To improve both the confidence of the keyworkers and more effectively and safely manage service users in acute mental health crisis we put on several talks across two sites in the borough. These talks covered how to manage, risk assess and appropriate referral to the various pathways within the North London Mental Health Partnership; the roles of Mental Health Crisis Assessment Service (MHCAS), the crisis team and crisis house and when psychiatric admission may be merited.

**Results:** 30 keyworkers were surveyed before and after the talk on confidence in managing several domains of mental health crisis. Asked: How confident are you in managing a service user a. having a mental health crisis? b. with thoughts of self-harm? c. with suicidal thoughts? and finally d. Do you feel you know the appropriate next steps if you feel a patient is in mental health crisis?

There was an average increase amongst key workers for a. 27%, b. 26%, c. 25% and d. 30% following the talk.

**Conclusion:** Presentations from clinicians are an effective way of improving the confidence of nonmedical substance misuse keyworkers in managing service users in acute mental health crisis. This is likely to lead to more effective and safer management of community substance misuse service users in acute mental health crisis.

